# Supplementary material for: LRRK2 Inhibition Mitigates the Neuroinflammation Caused by TLR2-Specific α-Synuclein and Alleviates Neuroinflammation-Derived Dopaminergic Neuronal Loss
Source: Cells. 2022 Mar 2;11(5):861. doi: 10.3390/cells11050861 (PMC8909553; doi:10.3390/cells11050861)
Supplement: Supplementary file 1 [file cells-11-00861-s001.zip › cells-1575112-supplementary.pdf]

Supplementary Figures

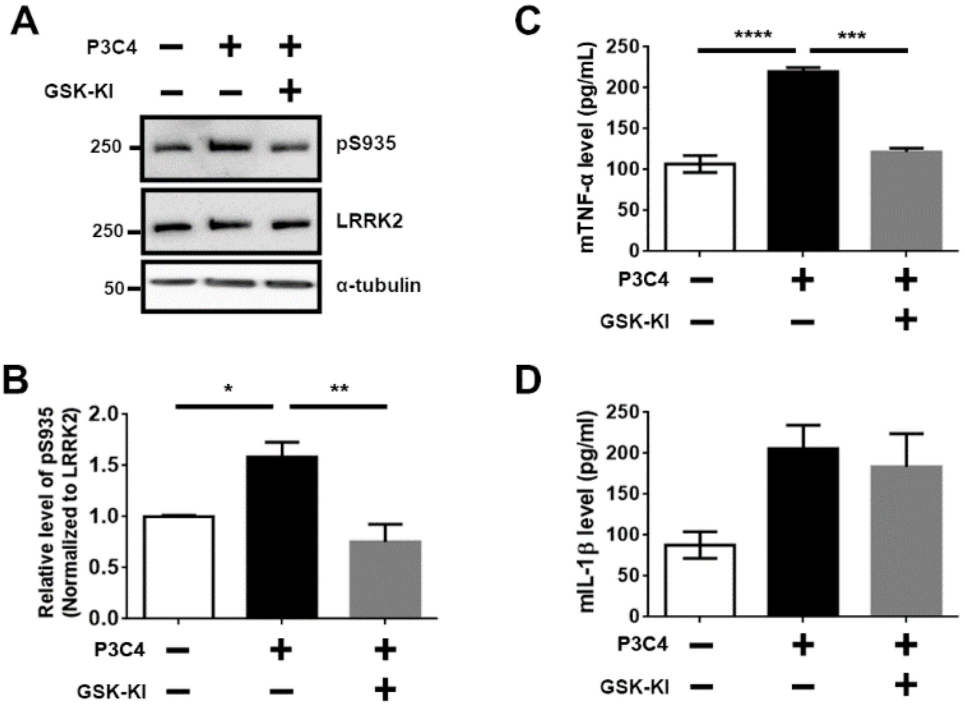

Figure S1. LRRK2 kinase inhibitor decreased TLR2-mediated upregulation of kinase activity and mTNFα level. (**A**,**B**) Pam3CSK4 (P3C4) treatment with or without GSK-KI (GSK2578215A) was subjected to western blot, and S935 phosphorylation levels of LRRK2 were analysed. We assessed the released levels of mTNFα (**C**) and mIL-1β (**D**) in the media from (A). One-way ANOVA with Bonferroni's post-hoc test, * $p < 0.05$, ** $p < 0.01$, *** $p < 0.001$, **** $p < 0.0001$; $n = 4$. Data are represented as mean ± SEM.

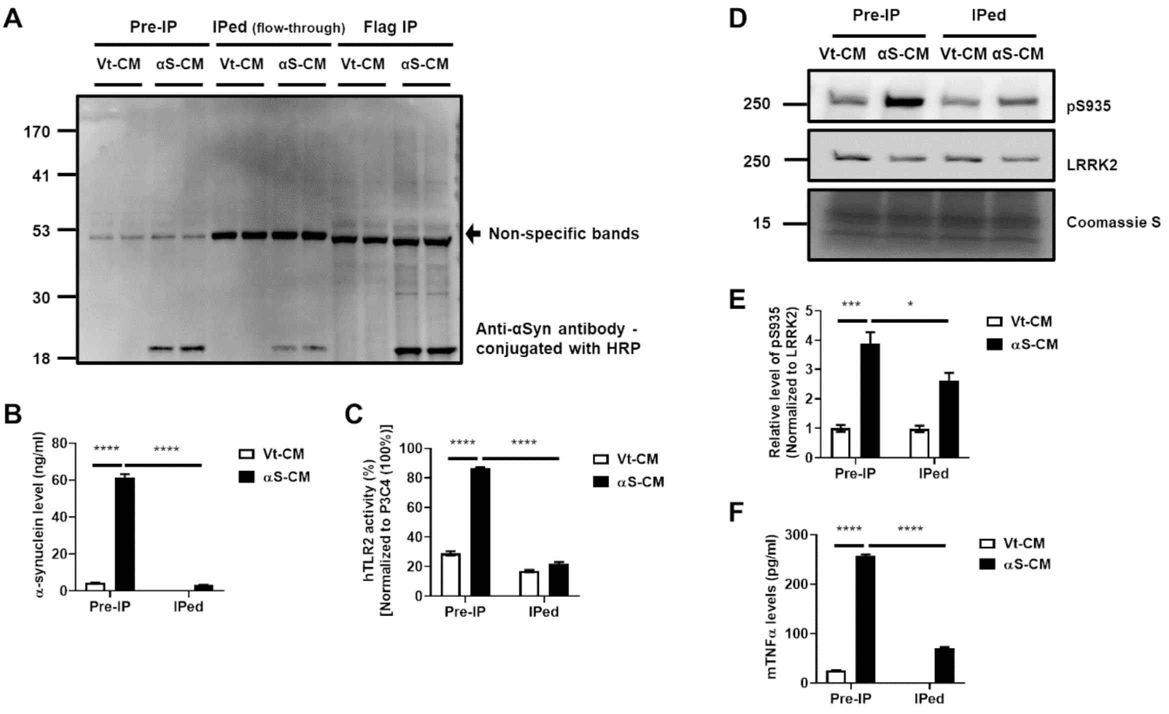

Figure S2. Removal of αSyn in αS-CM ameliorated LRRK2 kinase activation and pro-inflammatory cytokine releases in BV2 cells. The immunoprecipitation (IP) of original Vt- or αS-CM (Pre-IP) with Flag antibody segregated Vt- or αS-CM to Flag-IP fraction, the bead fraction, and IPed fraction, the supernatant of IP mixture. These fractions were analysed with western blot (**A**), αSyn sandwich ELISA (**B**), hTLR2 activity (**C**). The Pre-IP or IPed fraction were treated to BV2 cells. LRRK2 kinase activation (**D**,**E**), the levels of mTNFα release (**F**) were analysed. Student's *t*-test, * $p < 0.05$, *** $p < 0.001$, **** $p < 0.0001$; $n = 3$. Data are represented as mean ± SEM.

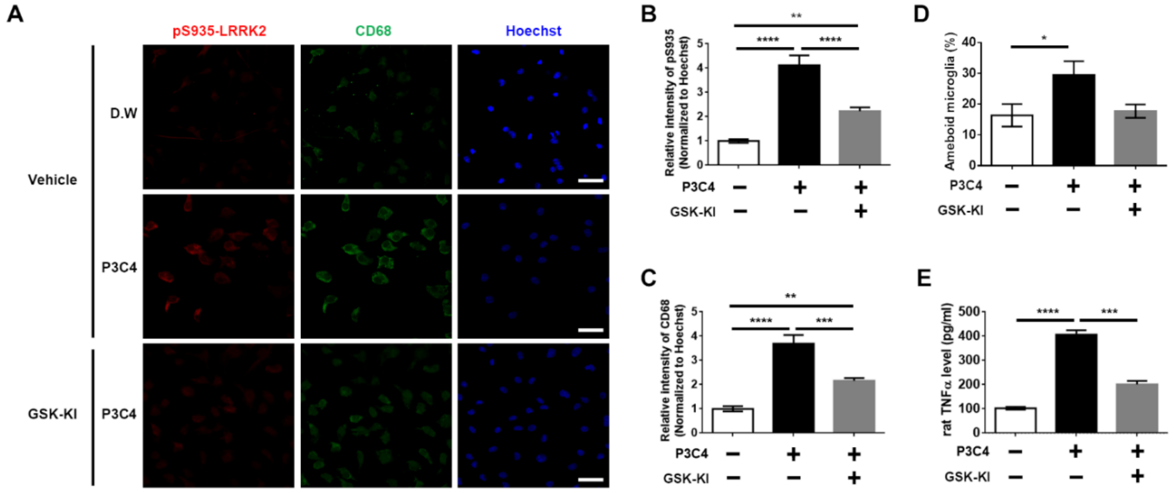

Figure S3. LRRK2 kinase inhibitor decreased TLR2-induced microglial activation in a rat primary microglia. The change of LRRK2 kinase activity by the treatment of P3C4 with or without GSK-KI was analysed with immunostaining of pS935 and CD68 (**A**–**C**), and the microglia with ameboid morphology were counted (**D**). Distilled water (D.W) was used for the solvent of P3C4. The released levels of rat TNFα from were measured by ELISA (**E**). n = 3, approximately 40-65 cells for IF. One-way ANOVA with Bonferroni's post-hoc test, * $p < 0.05$, ** $p < 0.01$, *** $p < 0.001$, **** $p < 0.0001$; n = 4. Data are represented as mean ± SEM.

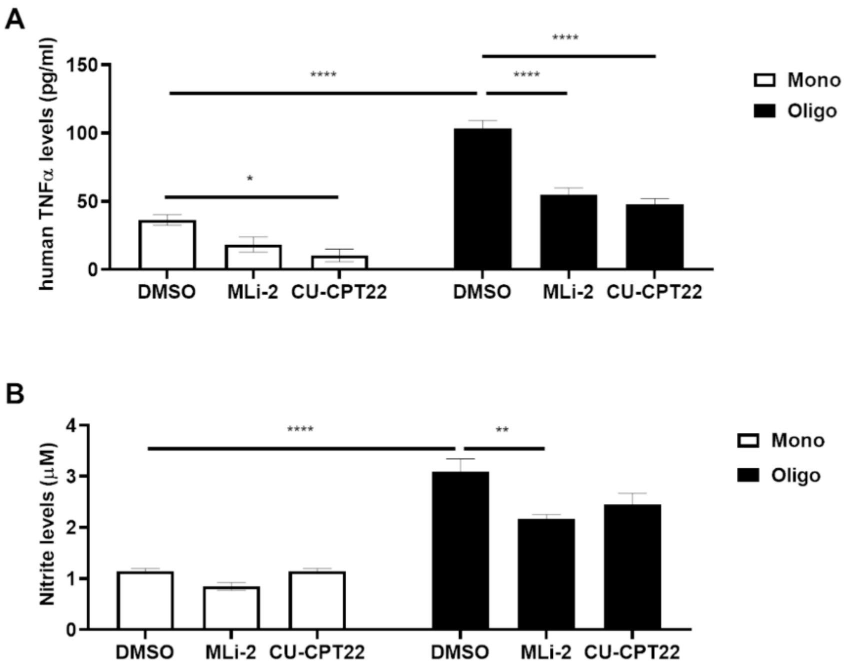

Figure S4. The increase of pro-inflammatory responses by oligomeric αSyn stimulating TLR2 was mitigated by the LRRK2 kinase inhibition and TLR2 antagonizing. The co-treatments of 0.2 μg/ml monomer αSyn (Mono) and oligomer (Oligo) with DMSO, MLi-2 (0.5 μM), or CU-CPT22 (0.5 μM), a TLR2 antagonist, were incubated for 18h in C20. The culture media were analysed with human TNFα ELISA (**A**) and Greiss assay (**B**). One-way ANOVA with Tukey's post-hoc test, * $p < 0.05$, ** $p < 0.01$, **** $p < 0.0001$; $n = 4$. Data are represented as mean ± SEM.

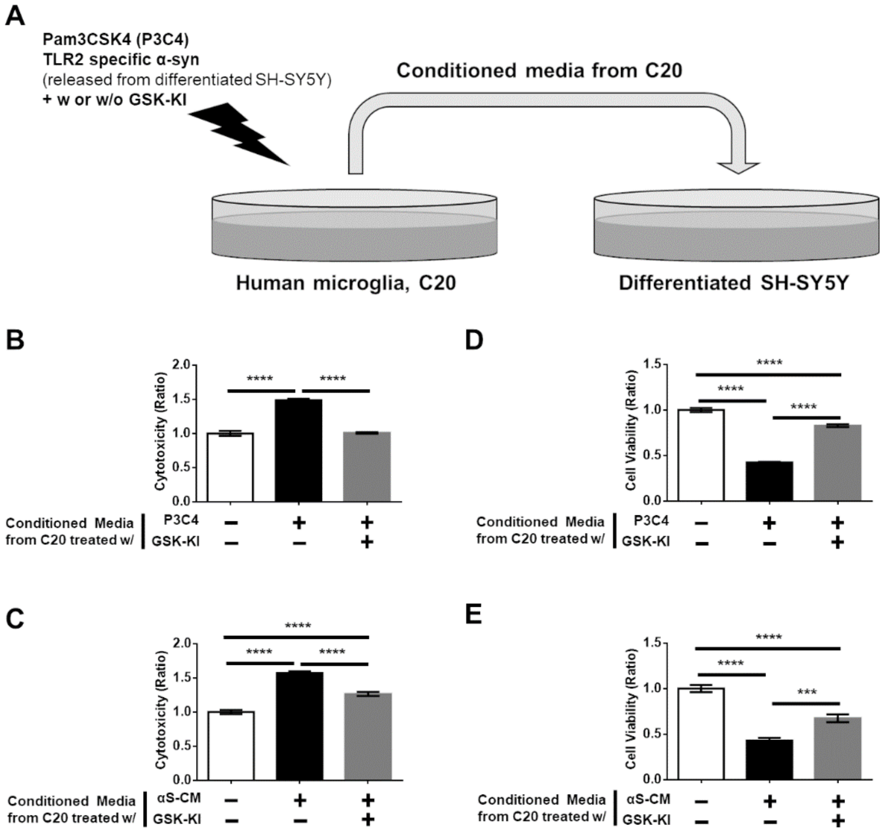

Figure S5. LRRK2 kinase inhibitor reduced the neuronal toxicity provoked by conditioned media of C20 via the TLR2 stimulation. The schematic description of the experiment was shown in (**A**). We measured the released LDH activity, as cytotoxicity (**B**), and cell viability using CCK-8 (**C**) in dSH cultured with the conditioned media from P3C4-treated C20. dSH cultured with the conditioned media from αS-CM-treated C20 was also analysed with cytotoxicity (**D**) and cell viability (**E**). One-way ANOVA with Tukey's post-hoc test, *** $p < 0.001$, **** $p < 0.0001$; $n = 5$. Data are represented as mean ± SEM.
